# Blinatumomab-induced remission followed by haploidentical transplantation in pediatric relapsed/refractory pre-B ALL: a multicenter study in Mexico

**DOI:** 10.3389/fonc.2025.1653329

**Published:** 2025-11-13

**Authors:** Alberto Olaya-Vargas, Haydee Salazar-Rosales, Yadira Melchor-Vidal, Martín Pérez-García, Annecy Herver-Olivares, Gerardo López-Hernández, Nideshda Ramírez-Uribe, Cesar Galván-Díaz, Irlanda Campos-Pérez, Pilar Cubria-Juárez, Angeles Del Campo-Martínez, Norma López-Santiago, Rocio Cárdenas-Cardos, Cesar Cárdenas-Pérez Gallardo, Ignacio Mora-Magaña, Jaime Shalkow-Klincovstein

**Affiliations:** 1 Department of Hematopoietic Stem Cell Transplantation and Cell Therapy, National Institute of Pediatrics, Mexico City, Mexico; 2 Department of Hematopoietic Stem Cell Transplantation and Cell Therapy, ABC Medical Center, Mexico City, Mexico; 3 Department of Hematopoietic Stem Cell Transplantation and Cell Therapy, Teleton Children’s Hospital for Oncology, Queretaro City, Mexico; 4 Department of Hematopoietic Stem Cell Transplantation and Cell Therapy, La Raza Medical Center, Mexican Social Security Institute (IMSS), Mexico City, Mexico; 5 Hemato-Oncology Service, National Institute of Pediatrics, Mexico City, Mexico; 6 Independent Statistical Advisor, Mexico City, Mexico

**Keywords:** relapsed/refractory acute lymphoblastic leukemia, haploidentical transplantation, blinatumomab, Latin America, pediatric cancer

## Abstract

**Background:**

Precursor B-cell acute lymphoblastic leukemia (pre-B ALL) is the most common pediatric cancer worldwide, with cure rates exceeding 85% in high-income countries. However, in *Mexico*, event-free survival (EFS) during first remission remains below 65%. Children with *relapsed or refractory CD19+ pre-B ALL* have dismal prognoses and limited therapeutic options. This study evaluated the efficacy and safety of *blinatumomab* as a bridge to allogeneic hematopoietic stem cell transplantation (HSCT), including *haploidentical HSCT*, in this high-risk population.

**Methods:**

This multicenter, prospective interventional study was conducted between February 2017 and December 2022 across four pediatric oncology centers in Mexico. Fifty-four patients (aged 8 months to 18 years) with refractory or high-risk relapsed CD19+ pre-B ALL received one or two cycles of *blinatumomab*. Responders were consolidated with allogeneic HSCT. Primary endpoints included remission rates and clinical factors associated with response; secondary endpoints were blinatumomab-related toxicity, overall survival (OS), EFS, and transplant outcomes.

**Results:**

Among 54 patients, 24 (44.5%) received one cycle, achieving a molecular complete remission rate of 75%. Thirty (55.5%) received two cycles, with 73.3% reaching deep remission (p = 0.89). Forty-one patients (76%) proceeded to HSCT, including 70% who received *haploidentical transplants*. Thirteen did not undergo HSCT due to refractory disease (n = 8) or partial response (n = 5). At 60 months, transplanted patients achieved an OS of 82% and EFS of 68%, with the best survival in matched related donor recipients (100%) versus haploidentical (58%). *Blinatumomab* demonstrated a favorable safety profile with no treatment-related mortality.

**Conclusion:**

Blinatumomab followed by haploidentical HSCT offers an effective and feasible therapeutic strategy for *relapsed or refractory CD19+ pre-B ALL* in *Mexico*, improving long-term survival in resource-limited settings.

## Introduction

Precursor B-cell acute lymphoblastic leukemia (pre-B ALL) is the most common malignancy in children and typically responds well to initial therapy, with remission rates of 80–90%, and long-term survival exceeding 90% in high-income countries (1, 2). However, outcomes are significantly worse in relapsed or refractory cases, with long-term remission rates falling to 30–40% (3). In Mexico, event-free survival (EFS) during first remission is reported to be no greater than 65%, underscoring the critical need for more effective therapeutic strategies, particularly in resource-limited settings (4–6).Allogeneic hematopoietic stem cell transplantation (HSCT) remains a cornerstone of treatment for patients with relapsed or refractory disease. Nevertheless, finding a suitable HLA-matched donor is frequently challenging. In this context, haploidentical HSCT has emerged as a viable alternative, especially in regions with limited access to unrelated donorregistries (7). Achieving remission assessed by minimal residual disease (MRD) by flow cytometry before HSCT—specifically, (MRD) levels below 0.01%—has been consistently associated with improved post-transplant outcomes (7, 8).

Blinatumomab, a bispecific T-cell engager targeting CD19 and CD3, has demonstrated efficacy in inducing MRD-negative remission in children with relapsed or refractory CD19+ pre-B ALL. Compared to conventional chemotherapy, it offers lower systemic toxicity, a targeted mechanism of action, and serves as an effective bridge to transplantation (9).

This study aimed to evaluate the efficacy and safety of one or two cycles of blinatumomab for inducing molecular remission in pediatric patients with relapsed or refractory CD19+ pre-B ALL, followed by consolidation haploidentical HSCT. We present the outcomes of a multicenter experience from Mexico, and discuss its implications for treatment protocols in similar settings.

## Materials and methods

This multicenter, interventional, non-randomized study with single-group assignment, was conducted between February 2017 and December 2022 across four institutions in Mexico. The protocol was accepted by the respective IRB at all four participating institutions. Eligible participants were children aged 0 to 18 years with relapsed or refractory CD19-positive pre-B acute lymphoblastic leukemia (ALL), and a Lansky performance status score above 80. Patients with isolated extramedullary relapse were excluded.

### Baseline assessment and eligibility

Upon enrollment, all patients underwent immunophenotyping to confirm CD19 expression in leukemic blasts. HLA class I and II compatibility were assessed using PCR-SSP techniques at the Hemato-Oncology Laboratory to facilitate donor selection. Evaluation of central nervous system involvement was performed via lumbar puncture. Baseline assessments also included screening for extramedullary disease and comorbidities.

### Chemotherapy and blinatumomab administration

All patients received a tumor debulking chemotherapy regimen, consisting of an anthracycline, asparaginase, vincristine, and corticosteroids at their respective institutions, prior to initiating blinatumomab. Blinatumomab was administered as a continuous intravenous infusion: 5 µg/m²/day for the first 7 days, followed by 15 µg/m²/day for the next 21 days. Intrathecal triple therapy with methotrexate, cytarabine, and hydrocortisone was administered two weeks after each blinatumomab cycle. All cycles were delivered in an inpatient setting, and toxicity was monitored daily.

### Transplant conditioning and Graft Versus Host Disease prophylaxis

Patients achieving remission—defined as MRD < 0.01%—following one or two blinatumomab cycles, proceeded to allogeneic hematopoietic stem cell transplantation (HSCT). The conditioning regimen consisted of total body irradiation (TBI; 200 cGy/day on days –7 to –5), cyclophosphamide (60 mg/kg/day on days –4 and –3), and etoposide (1,200 mg/m² on day –2). GVHD prophylaxis included post-transplant cyclophosphamide (60 mg/kg on days +3 and +4), tacrolimus, and mycophenolate mofetil.

All patients were evaluated prior to transplantation to exclude organ dysfunction and to optimize peri-transplant safety. Informed consent was obtained from parents or legal guardians, as well as from patients aged ≥9 years. Donors also received counseling and provided informed consent.

### Stem cell mobilization and quality control

Donors received granulocyte-colony stimulating factor (G-CSF) at 10 µg/kg/day for four days (two doses on day 4). Peripheral blood stem cells were collected via leukapheresis using the COBE Spectra 7.0 Apheresis System on day 5. Cell product quality was assessed using flow cytometry (FACS CANTO, Becton Dickinson), quantifying CD34+, CD3+, CD19+, and CD45+ viable cells according to ISHAGE guidelines (10). Neutrophil engraftment was defined as an absolute neutrophil count >500 cells/µL for three consecutive days, platelet engraftment was considered with a platelet count >20 x 10^9^, for 7 consecutive days without transfusion support.

### Post-transplant monitoring

Post-HSCT follow-up included serial chimerism analysis (complete chimerism ≥95%) and immune reconstitution profiling at 3, 6, 12, and 18 months. Flow cytometry was used to evaluate CD3+, CD4+, CD8+, CD19+, and CD16 + 56+ populations. Viral loads for cytomegalovirus (CMV) and Epstein–Barr virus (EBV) were monitored using RT-PCR weekly for 6 months, biweekly up to 12 months, and monthly thereafter.

### Statistical analysis

Descriptive statistics were used to summarize baseline characteristics. Bivariate analyses were performed using Chi-square and Student’s t-tests. Overall survival (OS) and event-free survival (EFS) were estimated using Kaplan–Meier curves, and compared with the log-rank test. Subgroup analyses evaluated outcomes according to pre-blinatumomab leukocyte counts (<10,000/μL, 10,000–50,000/μL, >50,000/μL), remission status, MRD, cell dose, age, sex, and donor type. A p-value of <0.05 was considered statistically significant. Analyses were conducted using SPSS software version 20.0.

## Results

### Patient characteristic

A total of 54 patients from four institutions (three in Mexico City and one in Querétaro) were included. The cohort was evenly distributed by sex (27 female, 27 male), with ages ranging from 8 months to 18 years. Age distribution was as follows: 6% were younger than 1 year, 64% were between 1 and 10 years, and 30% were older than 10 years. (See **Table 1**).

**Table 1:** Patient characteristics.

Characteristic	N (%)
Sex (Female/Male)	27 (50%) / 27 (50%)
Age <1 year	3 (6%)
Age 1–10 years	35 (64%)
Age >10 years	16 (30%)
First high-risk relapse	35 (65%)
Second relapse	9 (16%)
Refractory disease	10 (18%)
WBC <10,000/μL	42 (78%)
WBC 10,000–50,000/μL	10 (18.5%)
WBC >50,000/μL	2 (3.6%)

WBC, White Blood Cells.

Regarding disease status, 65% (n = 35) had a first high-risk relapse according to the BFM criteria (11, 12), 16% (n = 9) had a second relapse, and 18% (n = 10) had refractory disease (Defined as those patients who, after a complete second-line induction regimen, had hematological recovery with blasts confirmed by flow cytometry) (12). The pre-treatment leukocyte count ranged from 1,400 to 162,000 cells/μL, with a median of 8,415/μL. Leukocyte count distribution was: <10,000/μL in 78% (n = 42), 10,000–50,000/μL in 18.5% (n = 10), and >50,000/μL in 3.6% (n = 2).

### Response to blinatumomab

Among the 54 patients, 24 (44.5%) received a single cycle of blinatumomab. Of these, 75% (n = 18) achieved complete remission, while 25% (n = 6) exhibited a partial response or remained refractory. Overall, 33.3% (n = 18) of the total cohort achieved deep remission after one cycle. (See **Table 2**).

**Table 2:** Treatment response and prognostic factors.

Variable	Outcome	P-value
1 cycle (n=24)	18 (75%) CR; 6 (25%) NR/PR	0.89
2 cycles (n=30)	22 (73.3%) CR; 8 (26.6%) NR	0.89
Total Responder/Non-Responder Patients (n54)	40(74%) Responder 14(26%) Non Responder	
>1 prior relapse/refractory	OR 6.3 (failure)	NS
Age >10 years	OR 4.6 (failure)	0.038, OR: 9.11; 95% CI: 1.12–73.66
Female sex	OR 0.15 (less likely CR)	0.09
≥2 prior chemo lines	OR 0.125 (lower CR)	0.08

NS, Non-statistical significance.

The remaining 30 patients (55.5%) received two cycles, with 73.3% (n = 22) achieving deep remission. The remaining 26.6% (n = 8) showed no response. There was no statistically significant difference in complete remission rates between those who received one versus two cycles (75% vs. 73.3%, χ² = 0.0193, p = 0.89).

### Prognostic factors for treatment response

Several clinical variables correlated with treatment outcomes:

Patients with more than one prior relapse or with refractory disease were 6.3 times more likely to fail achieving remission, although this was not statistically significant. Patients older than 10 years were 4.6 times more likely to fail to achieve remission. Female patients had a 6.7-fold lower likelihood of achieving complete remission compared to males (p = 0.09). (See **Table 2**).

Receiving two or more prior lines of chemotherapy reduced the odds of remission by 8-fold (p = 0.08).

Given the limited sample size, a p-value threshold of <0.05 statistics, p-values ​​>0.05 to 0.1 were used for exploratory purposes. Under this criterion, all these associations were considered suggestive.

### Multivariate analysis

A logistic regression was performed for the dependent variable “Remission after Blinatumomab.” After adjustment for potential confounders:

Receiving two or more cycles of blinatumomab was associated with a 41% increase in the odds of remission, although this was not statistically significant.

Patients older than 10 years had a significantly increased likelihood of treatment failure (OR = 9.11, 95% CI: 1.12–73.66; p = 0.038).

Prior exposure to two or more lines of chemotherapy significantly reduced remission probability (OR = 0.09, 95% CI: 0.01–0.99; p = 0.049) (See **Table 3**).

**Table 3:** Multivariate logistic regression.

Variable	OR	95% CI	P-value
≥2 blinatumomab cycles	1.41	—	NS
Age >10 years	9.11	1.12–73.66	0.038
≥2 chemo lines Previus	0.09	0.01–0.99	0.049

NS, Non-statistical significance.

### Toxicity profile

Grade I–II cytokine release syndrome (CRS) was observed in 53.6% of patients, typically presenting as fever and tachycardia. One patient developed Grade III CRS, and in 2 patients grade IV was present, one with pleural effusion and cardiac failure, neurologic toxicity occurred in one patient, who experienced seizures during the first cycle; symptoms resolved with supportive management. One patient with refractory disease died from gram-negative sepsis during treatment. (See **Table 4**).

**Table 4:** Toxicity profile [graded by CTCAE v5.0; CRS by ASTCT (23); neurotoxicity by ICANS (24)].

Toxicity	N (%)
Grade I	25 (46.3%)
Grade II	4 (7.3%)
Grade III	1 (2.4%)
Grade IV	2 (4.8%)
No toxicity	21 (39%)

23. Biology of Blood and Marrow Transplantation, Volume 25, Issue 4, 625 – 638.

24. Blood Adv. 2020 Apr 14;4(7):1440-1447.

### Hematopoietic stem cell transplantation outcomes

A total of 41 patients (76%) underwent HSCT, while 13 (24%) did not—primarily due to refractory disease (n = 8) or failure to achieve complete remission (n = 5).

Of those transplanted:

38 patients (70%) received haploidentical HSCT, 3 patients (6%) received fully HLA-matched related donor transplants. The median CD34+ cell dose was 5.9 × 10^6^/kg. Neutrophil engraftment was achieved in all patients between days +12 and +16, while platelet engraftment occurred between days +14 and +21, only 2 patients presented unstable chimera associated with BK virus infection, in both cases donor lymphocyte infusion (DLI) was used to successfully recover the graft. No transplant-related mortality occurred within the first 100 days.

GVHD incidence:

- Acute GVHD (aGVHD): 36% (n = 15), 9% (n 4) grade I, 10%(n 5) grade II, 17%(6) grade III, no patient presented grade IV,31% (n 13) were present in the haploidentical model and 5% (n 2) in the HLA-identical related donor model. There were no deaths related to aGVHD.
- Chronic GVHD: 41% (n = 17), 27% (n = 11) presented a moderate or severe form, 14% (n = 6) had a mild form, the survival of patients who presented cGVHD was 88%, with a mortality of 17.6% (n = 3) at 5 years.
- The leading cause of death post-HSCT was disease relapse (See **Table 5**).

**Table 5:** HSCT and survival outcomes.

Outcome	Value 95%. CI
Underwent HSCT	41 (76%) p>0.0001% OS (82%. CI: 60-96 %
No HSCT	13 (24%) OS (0%)
Haploidentical HSCT	38 (70%)
Fully matched donor	3 (6%)
Acute GVHD	15 (36%)
Chronic GVHD	17 (41%)
OS at 60 months (Blinatumomab + HSCT)	82%
EFS at 60 months	68%
OS by CR status (CR)	66.7% p>0.01%
OS by CR status (PR)	18% CI:50.3-84.4%
OS by CR status (NR)	0%
OS by donor type (HLA-Identical Related Donor)	100% NS
OS by donor type (Haplo)	58%

AHSCT, Hematopoietic Stem Cell Trasplantation; GVHD, Graft Versus Host Disease; OS, Overall Survival. CR, Complete Remission; PR, Partial Remission; NR, No Remission; NS, Non-statistical significance.

### Survival outcomes

**Figure 1:**
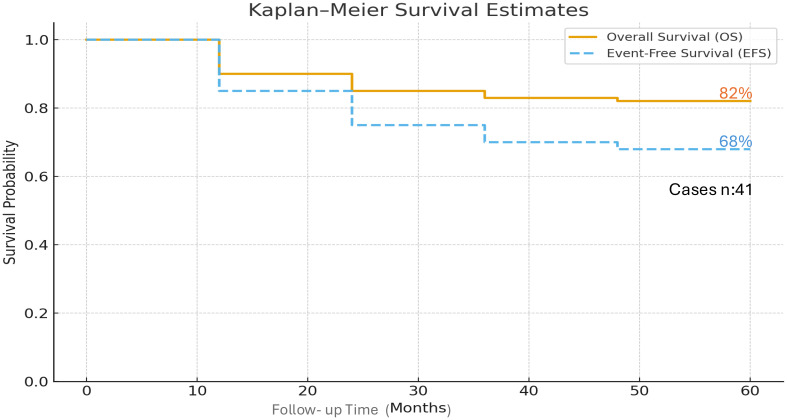
Overall Survival and Event-Free Survival at 60 months of 41 pediatric patients with relapsed/refractory ALL who received blinatumumab as therapy to achieve remission and allogenic hematopoietic cell transplantation.

At 60 months, overall survival (OS) in patients who received blinatumomab followed by HSCT was 82%, event-free survival (EFS) was 68%. (See **Figure 1**).

**Figure 2:**
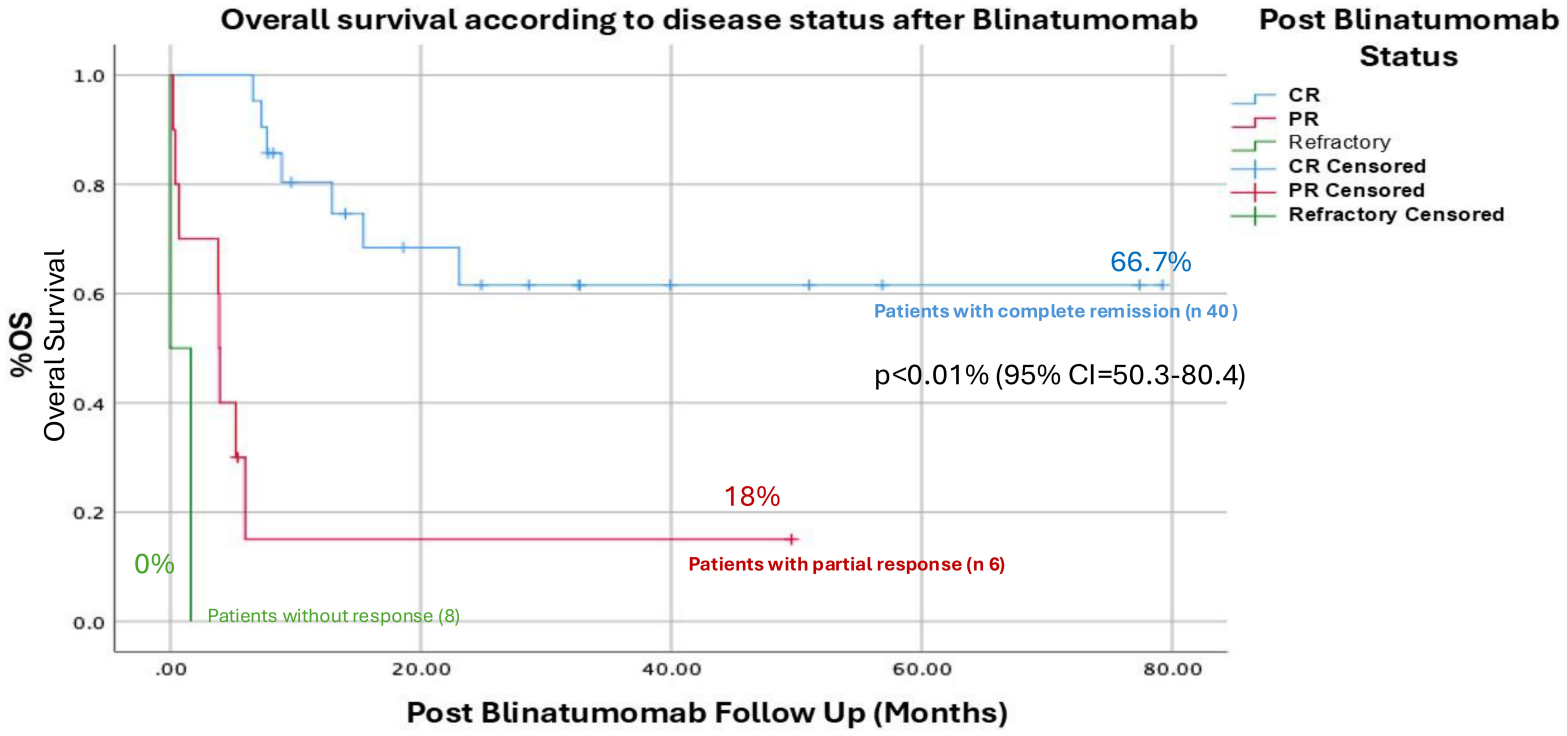
Overall Survival according to the response after administration of Blinatumomab, the difference between patients with complete response (OS 66.7%) compared to patients who obtained a partial response (18%) and no response (0%) was significant p<0.01% (95% CI =50.3-80.4%) for patients with complete remission.

Survival by response status:

- Complete remission: 66.7%.
- Partial remission: 18%.
- No response: 0% (See **Figure 2**).

**Figure 3:**
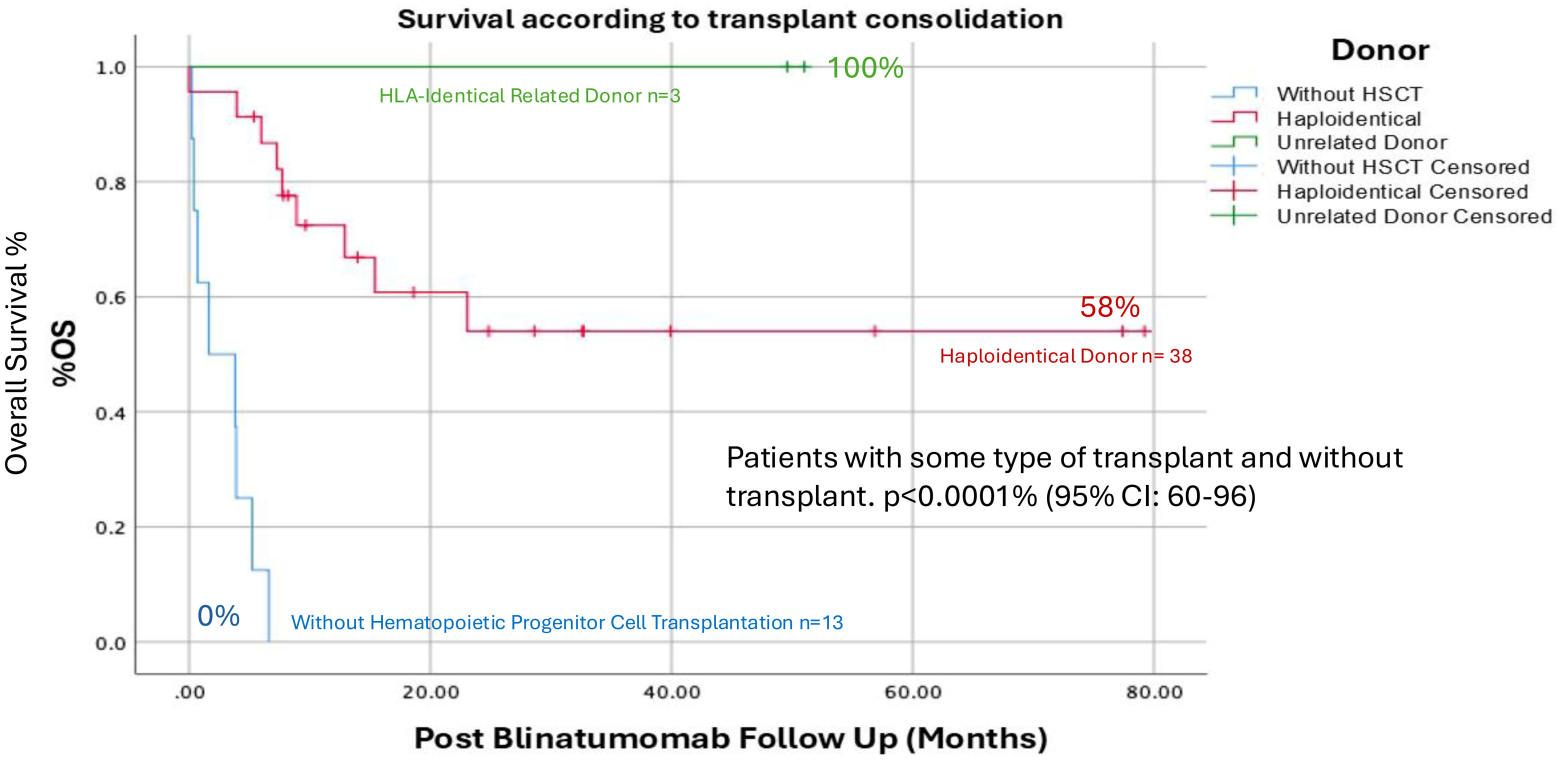
Overall survival by type of transplant received after obtaining remission with Blinatumumab, apparently the transplants performed with an HLA-identical family donor presented a GS of 100%, however the n was only 3 patients, where we did find a statistically significant difference p<0.001% between the group of patients who received some type of transplant and those who were not transplanted. (CI: 60-96%).

Survival by donor type:

- Fully matched related donor: 100%.
- Haploidentical donor: 58% (See **Table 5**).
- It appears preferable to have a 100% compatible related donor; these data should be taken with caution because the population for this type of transplant was very limited. Patients with haploidentical transplants were mostly used due to the lack of routine access to unrelated donors in Mexico, and this may explain the higher frequency of association with mortality-related complications such as GVHD, infections, and relapse. (See **Figure 3**).

## Discussion

Relapsed or refractory (R/R) pre-B acute lymphoblastic leukemia (ALL) continues to represent a major clinical challenge in Mexico and across Latin America. Despite advances in first-line therapies, over 35% of pediatric patients in our setting experience early high-risk relapses, often associated with underlying adverse cytogenetic or molecular profiles, such as “triple-hit” leukemias. Traditional salvage regimens relying on intensive chemotherapy have demonstrated cure rates below 20%, and are associated with considerable toxicity and treatment-related mortality (13).

Our study demonstrates that blinatumomab is effective in inducing complete remission in children with R/R CD19+ pre-B ALL, with 75% of patients achieving remission after a single cycle, and 73.3% after two cycles, although the difference between the groups was not significant, this could be due to the fact that patients with a greater number of lines of prior chemotherapy treatment and older > 10 years of age were concentrated in the group that received 2 cycles, only 2 patients who had achieved a partial response with the first cycle achieved a complete remission with the 2nd cycle. These findings are consistent with international reports, such as the RIALTO (14) and ALCANTARA trials (15), which reported similar remission rates in pediatric and adult cohorts, respectively.

Interestingly, no significant difference was observed in remission rates between one and two cycles, suggesting that a single cycle may be sufficient for most patients. However, the higher proportion of deep remissions observed after two cycles supports their use in patients with suboptimal initial response or delayed clearance of disease.

We identified several clinical predictors associated with a lower likelihood of remission:

- Age >10 years.
- History of multiple relapses or refractory disease.
- Exposure to ≥2 previous lines of chemotherapy.

These findings align with prior studies, including Locatelli et al (14, 16), which noted poorer blinatumomab responses in adolescents and young adults. Our multivariate analysis confirmed age and prior chemotherapy exposure as significant negative predictors, emphasizing the importance of early intervention with immunotherapy in this population.

Of note, female sex was associated with lower remission rates, though this did not reach conventional statistical significance. This observation warrants further investigation in larger cohorts.

Blinatumomab was generally well tolerated. The most common adverse event was Grade I–II cytokine release syndrome (CRS), occurring in 65% of patients. This incidence is consistent with previous pediatric studies reporting 50–70% rates of low-grade CRS (16). Only one patient developed Grade IV CRS, and neurologic toxicity was limited to a single case of reversible seizures.

Importantly, the low toxicity profile allowed many patients to recover from prior chemotherapy-related complications, improve nutritional status, and remain clinically stable during therapy—factors that may facilitate successful transition to transplant. Although not formally analyzed, these supportive benefits were frequently noted during clinical observation (17).

The integration of HSCT following blinatumomab-induced remission significantly improved survival, with a 5-year overall survival rate of 82% and an Event-Free Survival (EFS) of 68% among transplanted patients. Among these, all patients who received a fully matched related donor transplant survived, while survival in the haploidentical group was 58%. These outcomes are comparable to those reported in the RIALTO study, which documented 5-year OS rates ranging from 60% to 80% depending on donor type and disease status (14).

While the superior survival in matched donor recipients is noteworthy, it is critical to emphasize the limited availability of matched donors in Mexico. Thus, haploidentical transplantation represents a practical and accessible alternative that still confers meaningful survival benefit. None of the non-transplanted patients survived beyond follow-up, reinforcing the necessity of transplant as a consolidative strategy.

The results of this study support a sequential approach involving blinatumomab to induce remission, followed by haploidentical HSCT. This strategy is particularly valuable in low- and middle-income countries, where donor availability is limited, and toxicity from chemotherapy regimens poses additional risks.

Moreover, a cost-effectiveness analysis conducted in Mexico by Díaz-Martínez et al. (18) demonstrated that, beyond its clinical benefits, blinatumomab reduces costs associated with relapse-related hospitalizations, ICU admissions, and therapeutic failure. These findings further justify its incorporation into national treatment protocols.

Our study suggests that access to blinatumomab alone is insufficient—survival benefit is only observed when followed by HSCT. Therefore, immunotherapy programs must be linked to transplant services to ensure favorable long-term outcomes. Survival in non-transplanted patients: 0% (all died from disease progression).

Blinatumomab, has transformed the management of relapsed or refractory B-cell acute lymphoblastic leukemia (B-ALL) by inducing deep remissions and enabling subsequent hematopoietic stem cell transplantation (HSCT). However in our study 14 patients (26%) did not respond to blinatumomab. The Blinatumomab resistance can emerge through several mechanisms. The most prominent is antigen escape, including loss or alternative splicing of the CD19 epitope and lineage switch to myeloid or mixed phenotype acute leukemia, particularly in KMT2A-rearranged cases. T-cell dysfunction, driven by PD-1/PD-L1 immune checkpoint upregulation and exhaustion during continuous infusion, can also impair cytotoxicity. Additionally, high tumor burden, an immunosuppressive microenvironment, and sanctuary sites such as the central nervous system contribute to reduced efficacy (19, 20).

When resistance occurs, therapeutic strategies depend on the relapse biology and clinical context. Inotuzumab ozogamicin (anti-CD22 antibody–drug conjugate) is effective even in CD19-negative disease and often serves as a bridge to allogeneic HSCT. CAR-T cell therapy remains an option if CD19 expression is retained, while CD22-targeted or dual-target (CD19/CD22) CAR-T products are under investigation for CD19-negative relapse. Immune checkpoint inhibitors combined with blinatumomab are being explored to overcome T-cell exhaustion. Comprehensive re-evaluation of immunophenotype and genetics at relapse is crucial to guide therapy selection, and enrollment in clinical trials investigating novel bispecific T-cell engagers or next-generation CAR-T platforms is recommended (21, 22). Therefore, it is important that Mexico and Latin America continue working to provide access to new therapies such as CARS-T (22).

Neurological toxicity associated with blinatumomab is a known, generally reversible adverse effect that may include headache, tremor, altered mental status, and, less frequently, seizures. In our study, only one patient experienced seizures attributable to blinatumomab; the event was successfully managed with anticonvulsant therapy (levetiracetam). Once the seizures resolved, blinatumomab was reinitiated without recurrence of neurological symptoms, and the anticonvulsant treatment was discontinued six months later without further complications (23, 23).

The main limitation of the study was a small sample size, however in the context of Latin America the results reported in this study are useful for decision-making in our countries.

## Conclusion

Blinatumomab represents a significant advancement in the treatment of relapsed or refractory CD19-positive pre-B acute lymphoblastic leukemia in children. Our findings demonstrate that blinatumomab is both effective and well tolerated as a bridge to allogeneic hematopoietic stem cell transplantation (HSCT), achieving high molecular remission rates with manageable toxicity. When followed by HSCT—including haploidentical transplantation in the absence of fully matched donors—this strategy leads to encouraging long-term survival outcomes, even in resource-limited settings.

In Mexico, where access to fully matched donors is often constrained and traditional chemotherapy regimens carry a high burden of toxicity, the integration of immunotherapy and haploidentical transplantation offers a feasible, cost-effective, and potentially life-saving approach for pediatric patients with relapsed or refractory ALL.

These results support the inclusion of blinatumomab-based strategies in national treatment protocols, and highlight the need for coordinated access to both immunotherapy and transplant services. Future prospective studies are warranted to confirm these findings, and optimize treatment sequencing and patient selection criteria.

## Data Availability

The raw data supporting the conclusions of this article will be made available by the authors, without undue reservation.
